# Unmasking the Enigma of the COVID Toe

**DOI:** 10.7759/cureus.48641

**Published:** 2023-11-11

**Authors:** Shirisha Saripalli

**Affiliations:** 1 Medicine, County Durham and Darlington National Health Services (NHS) Trust, Darlington, GBR

**Keywords:** sequelae of covid, systemic steroids, covid antibodies, ace inhibitors and covid-19, covid toe

## Abstract

In 2020, the world encountered a new infection known as COVID-19. As a result, people started experiencing various COVID manifestations, aftereffects, and sequelae, one of which is the COVID toe, a rather uncommon presentation. Chilblain-like lesions, commonly referred to as 'COVID toe' due to their high occurrence on toes and fingers, can develop in any age group but are most commonly seen in younger adults.

A 35-year-old female presented with painful pinkish-purple discoloration of her toes a week after acquiring a COVID-19 infection. Following this, she underwent blood tests and scans to rule out vasculitis and other autoimmune illnesses. The case highlights the distinctive presentation of this condition and its unusual appearance. This emphasizes that the COVID toe can vary from person to person, and not all cases are self-limiting. Depending on the presentation and the duration of symptoms, one may need to consider more extensive treatment, as not all cases resolve by themselves. This case report was presented as a poster at the Acute and General Medicine Conference in London, United Kingdom.

## Introduction

Chilblain-like lesions, often called 'Covid toe,' are cold-induced skin conditions characterized by erythematous or violaceous discolorations. These lesions are frequently accompanied by symptoms such as itching, swelling, discomfort, burning, blistering, or ulceration [1]. Research indicates that the occurrence of this condition is influenced by various factors, including age, gender, race, and geographical location.

Children are more susceptible to chilblain and chilblain-like lesions, with females being twice as likely to experience them as males. Caucasians are at a higher risk compared to individuals of other racial backgrounds, and the hands and feet are the most commonly affected areas. Geographical location also plays a pivotal role, with the majority of reported cases in Europe and North America, while cases in Asia are comparatively scarce. This is supported by studies conducted during the initial wave of the pandemic in China, where such lesions were not observed, and research in North America, which revealed that individuals with darker skin had very few cases. These findings underscore the significant influence of both race and geography on the development of chilblain lesions [2]. Interestingly, since the onset of the COVID pandemic, there have been instances of patients exhibiting symptoms that appear unrelated to cold weather.

## Case presentation

A 36-year-old woman tested positive for COVID-19 antibodies while ill with a viral infection. Her test for COVID-19 antigen was negative. Her past medical history included an uneventful pregnancy and vaginal delivery, and she had no previous co-morbidities. She had a family history (her grandfather) of lupus. Also, she smoked 10 cigarettes a day until three years ago and had 35 units of alcohol per week. She recovered from the illness with symptomatic treatment at home within a week. She did, however, notice the unpleasant discoloration of her left toes later.

**Figure 1:**
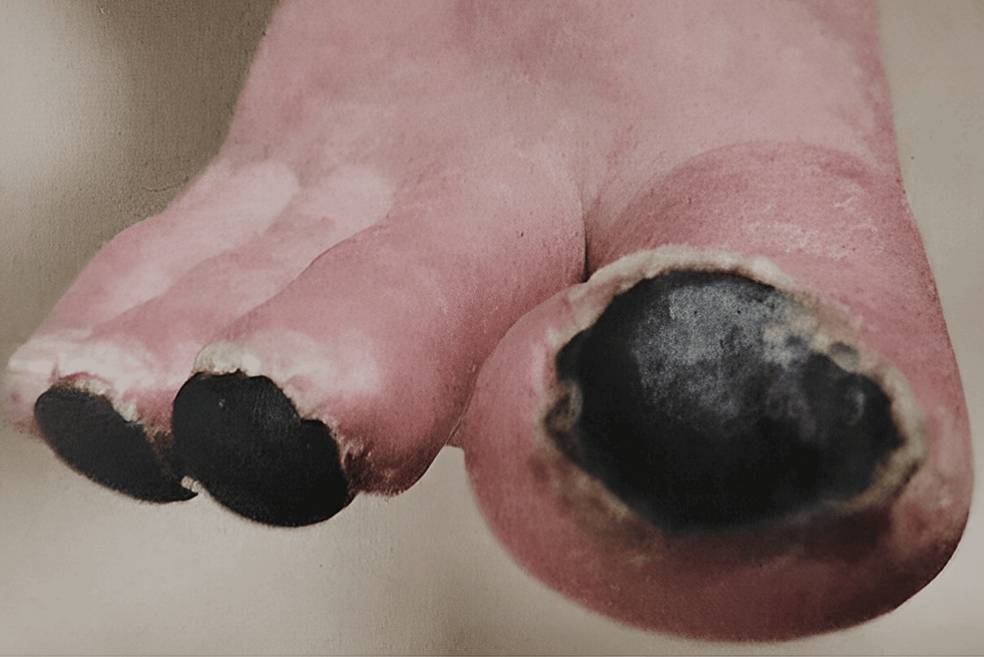
Black necrotic lesions on the tips of the patient's toes

**Figure 2:**
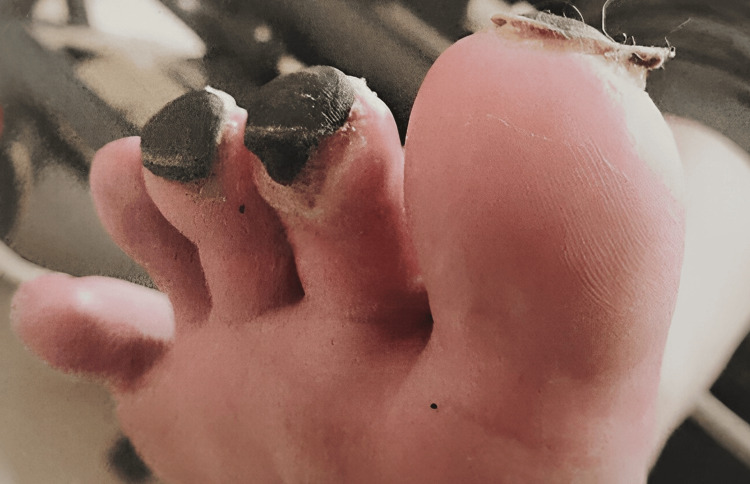
A clear demarcation is seen between the skin and the lesions. The second toe shows an area where the mass is set to fall off. The last two toes show that no scar tissue is left after healing.

The discoloration on the tips of her toes started as purplish and eventually turned into black necrotic masses, as seen in Figures 1-2. The vascular team conducted an additional assessment. Initial investigations by the vascular team included clotting factor testing and scans such as abdominal and lower limb ultrasound, contrast-enhanced CT abdomen, and CT positron emission tomography (PET) scans to rule out past conditions, all of which came back normal. She was initially given symptomatic treatment, which included non-steroidal anti-inflammatory drugs (NSAIDs) and a small dose of oral steroids. As she did not improve, she was hospitalized, started on an iloprost infusion, and intermittently switched to sildenafil. But as the lesions worsened, she was shifted back to iloprost. This treatment continued for 21 days, along with intermittent intravenous methylprednisolone. She was later discharged on prednisolone and analgesics, such as paracetamol and oxycodone, with the requirement of morphine as PRN considering the severe intensity of pain. The necrotic masses created a demarcation with the skin and eventually fell off without leaving any scars (Figure 2). But it took around six months for the symptoms to subside.

Following this major episode, she experienced three more viral infections. Subsequently, she had mild to moderate episodes characterized by discoloration with or without pain, and necrotic masses formed on her right toes. Additionally, it was recommended that she avoid COVID-19 vaccinations, as even a mild viral interaction could potentially trigger another condition.

## Discussion

The etiology is rooted in theories centered around two receptors: ACE and IFN-1. Using its spike protein, COVID-19 can bind to the ACE receptor found on eccrine sweat glands, the basal layer of the epidermis, and dermal blood vessels. Consequently, the effects may be primarily localized to the hands and feet, as these areas house the body's highest concentration of sweat glands. Notably, spike protein has been detected in chilblain-like lesion samples obtained from patients with suspected COVID toes, providing support for this theory [1]. Furthermore, the efficacy of the IFN-1 pathway in combating the infection is influenced by body temperature, as COVID-19 viral replication is significantly increased in cooler temperatures. This has led to the proposal that the IFN-1 pathway may be more effective in treating the infection in warmer environments, particularly when the virus is encountered via respiratory transmission [2].

Furthermore, multiple studies into the histopathology of COVID toes at 16 weeks after exposure have revealed that fibrin thrombi had occluded numerous papillary dermal capillaries, exhibiting endothelial cell swelling and a paucicellular inflammatory cell response, primarily a deep dermal infiltrate of small lymphocytes in and around eccrine coils. By using a direct immunofluorescence method, fibrin thrombi were found to stain faintly with periodic acid-Schiff (PAS+) and brightly with rabbit polyclonal anti-fibrin antibody [3]. Additionally, it has been hypothesized that young patients exhibit an early IFN-1 response, which can lead to the development of these lesions, in contrast to older and critically ill patients, who may experience an insufficient and delayed response. Also, they may have other conditions such as low vitamin D levels and hypercoagulopathy, which could contribute to acral ischemia due to coagulation disorders. Thus, COVID-19-induced chilblain-like lesions may in fact have a good prognosis [4].

A study conducted in Australia, based on a model that correlated temperature with the occurrence of lesions, revealed that as temperatures decreased, more lesions were observed. Additionally, other factors have been mentioned, including people moving around their houses barefoot during winter and exposing themselves to extreme cold, which could potentially explain the increasing number of cases during the pandemic [5].

Comparison with other studies

The majority of patients exhibit positive COVID-19 antibodies but negative COVID-19 test results, followed by the onset of toe discoloration after COVID-19 recovery [6]. With respect to differences, however, there is one notable distinction in our patient: she received extensive treatment, and her recovery period extended beyond that observed in other patients. This shows that such severe cases may benefit from low doses of steroids and analgesics to manage their condition. In contrast, other retrospective studies report recovery within a few weeks without the need for any medication [7]. This variation underscores the uniqueness of disease progression in each individual. Additionally, the accompanying images clearly demonstrate a distinct presentation of COVID toe featuring blackish-colored necrotic masses that are markedly different from other patients discussed in previous research.

Another treatment recommended by one of the studies is the use of cilostazol twice daily in patients suffering from COVID toe, possibly due to the presence of undiagnosed long COVID. Cilostazol has an impact on vessel tone, platelet activity, and rheologic blood flow, which has been shown to improve and expedite recovery. Furthermore, the study highlights the importance of testing for COVID-19 antibodies, even in cases where individuals may not have recent symptoms or a positive COVID-19 antigen test. In several instances, antibody tests have revealed elevated levels despite negative antigen results [8]. A few studies suggest that after the mRNA COVID-19 vaccination, some people began to report chilblain-like lesions on their hands and feet, with the major site being the feet. Therefore, avoiding vaccination for people who have contracted this condition could be a possible recommendation [9].

## Conclusions

This report explores the multifaceted factors influencing the development and progression of these lesions, including age, gender, race, and geographical location. The case was distinctive in terms of the extent of treatment required and the prolonged recovery period, and it also exemplified the individualized nature of disease progression in COVID toe, underscoring the importance of personalized care and management strategies. Comparing this case with other studies, it's clear that there is a common pattern of positive COVID-19 antibodies and negative test results preceding COVID toe symptoms. However, the severity and treatment response can vary among individuals, as illustrated by this case. The distinct presentation of black necrotic masses in this patient's condition highlights the heterogeneity of COVID toe manifestations and underscores the need for individualized evaluation and management. In conclusion, COVID toe remains a fascinating and complex dermatological phenomenon associated with the COVID-19 pandemic. Further research is needed to better understand the mechanisms behind its development and to refine treatment approaches for different presentations of the condition. Despite the infection's increasing occurrence since it first appeared, COVID toe is still a diagnosis that is a mystery in itself.
